# Adaptive control and state error prediction of flexible manipulators using radial basis function neural network and dynamic surface control method

**DOI:** 10.1371/journal.pone.0318601

**Published:** 2025-02-26

**Authors:** Yang Zhang, Liang Zhao

**Affiliations:** 1 Chongqing Preschool Education College, Wanzhou, Chongqing, China; 2 School of Information Engineering, Yangzhou University, Jiangsu, Yangzhou, China; Imperial College London, UNITED KINGDOM OF GREATBRITAIN AND NORTHERN IRELAND

## Abstract

This paper introduces a novel control strategy for managing the uncertainties in flexible joint manipulators, incorporating a Radial Basis Function Neural Network (RBFNN) with Adaptive Dynamic Surface Control (ADSC). This strategy innovatively utilizes RBFNN to precisely approximate uncertain system dynamics and integrates a nonlinear damping term to effectively counteract external disturbances, enhancing the overall control accuracy. We have also developed an adaptive law that updates neural network weights and system parameters in real-time, ensuring the system’s adaptability to dynamic changes. The application of the Lyapunov method ensures that all signals within the closed-loop system remain semi-globally uniformly bounded, significantly reducing tracking errors. Moreover, we introduce the use of Long Short-Term Memory (LSTM) networks for predictive analysis of state data, which further confirms the robustness and effectiveness of our control method through extensive simulations. The distinctive integration of these technologies and their practical validation through comparative simulations underscore the innovative aspects of our approach in addressing real-world challenges in flexible manipulators.

## 1 Introduction

A flexible robotic arm, typically featuring joints or structures made from bendable materials, is designed to navigate complex shapes and environments [1–3]. These arms are crucial for precise tasks like object grasping, flexible assembly, and collaborative operations, especially in constrained spaces such as medical surgeries, space exploration, and delicate material handling. Their adaptability and safety render them indispensable across a range of applications [4–6].

Investigations into the tracking control of mechanical systems have been shown to significantly boost automation, precision, and operational efficiency. These advancements not only broaden the range of potential applications but also lower both the costs and risks associated with operations. This is supported by the findings of [7,8], who discuss these improvements in their respective studies. Reference [9] explores robust tracking control for aircraft with actuator faults and damaged control surfaces, utilizing a multi-objective optimization strategy based on linear quadratic and $H_{\infty }$ performance metrics, validated through flight simulation examples. Li and Zhang et al. proposed two novel control strategies that effectively achieve precise trajectory tracking for quadrotor UAVs in the presence of dynamic obstacles and external disturbances, through the design of sliding mode disturbance observers and analysis using Lyapunov methods. Numerical simulation results validate the effectiveness of these control schemes [10]. In Reference [11], the study addresses asymptotic tracking control for uncertain nonlinear systems. Compared to traditional fuzzy adaptive control strategies [12,13], the integration of differential inclusion and set-valued mappings provides further theoretical validation for the controller’s local asymptotic tracking capabilities. This approach not only solidifies the theoretical base of the controller but also deepens our understanding of its precise tracking performance in a localized setting. A Naderolasli developed a new self-correcting adaptive strategy that significantly enhances the stability and control accuracy of two-degree-of-freedom tracking systems under external disturbances, parameter uncertainties, and measurement noise. This strategy utilizes inner and outer loop self-correcting stabilizers and trackers, along with an adaptive approach based on recursive least squares. The effectiveness of the proposed method has been validated through simulation techniques [14].

Moreover, uncertainty in system models can undermine the robustness of control systems, increasing their susceptibility to external disturbances and parameter variations, as noted in the references [15–17]. These uncertainties challenge the stability and performance of control systems, necessitating the implementation of robust [18,19] and adaptive control strategies [20,21] to maintain system effectiveness under varying conditions. Naderolasli A and Shojaei K et al. proposed a new controller for the formation of multiple autonomous surface vessels under the influence of model uncertainties and external disturbances, employing a leader-follower strategy and advanced control techniques to ensure formation stability. Simulation results validate the effectiveness and robustness of the control system [22]. In [23], a novel global regulator for flexible joint robots is proposed, which utilizes a nonlinear Proportional-Integral-Derivative (PID) control strategy. This regulator relies solely on motor position measurements and its global asymptotic stability is validated through closed-loop system analysis. Furthermore, Bounemeur and Chemachema proposed an adaptive fuzzy fault-tolerant tracking control method to address the challenges of multi-variable nonlinear systems with external disturbances, unknown control signs, and actuator faults. The method approximates the unknown nonlinear dynamics and state-dependent actuator faults using fuzzy logic systems and employs a Nussbaum-type function to tackle the issue of unknown control signs[24]. Reference [25] introduces a novel finite-time sliding mode control strategy for underwater robots, integrating backstepping and nonlinear disturbance observer techniques to address system uncertainties and time-varying disturbances. Simulation results demonstrate that this method enables robust signal tracking within a finite time frame, showcasing its effectiveness and disturbance rejection capabilities in practical underwater robotics applications. Reference [26] presents a robust adaptive formation control strategy for underactuated spacecraft using a neural network dynamic surface to manage external disturbances and uncertainties. Moreover, investigating the capacity of control systems, such as robots and drones, to operate effectively despite limitations on their outputs is critically important. Such research contributes to improving the systems’ efficiency, ensuring they operate as expected in restricted environments, thereby boosting their usability and dependability. Naderolasli A and colleagues developed a new leader-follower formation control method for Euler-Lagrange systems, optimizing system convergence rates and steady-state errors by employing an asymmetric barrier Lyapunov function and controlling trajectory tracking error boundaries. Simulation tests confirm the efficiency and stability of this control approach in dealing with unknown parameters and constrained system outputs [27]. Different from previous research by Naderolasli A, this study introduces a new constrained platoon formation controller design for autonomous underwater vehicles (AUVs) based on the dynamic surface control method, which effectively handles model uncertainties and field-of-view constraints. The tracking performance and safety are optimized through adaptive neural network technology. Simulation results confirm the high efficiency of this controller [28].

The Radial Basis Function Neural Network (RBFNN) uses radial basis functions as activation functions and is widely recognized for its proficiency in solving intricate nonlinear challenges. Bounemeur, chemachema, and others proposed an indirect adaptive fuzzy fault-tolerant control method that uses fuzzy systems to approximate uncertain nonlinearities and actuator faults, and employs a Nussbaum-type function to address the issue of unknown control gain sign. Simulation results validate the effectiveness of the approach, which overcomes the singularity problem in indirect adaptive feedback linearization control [29]. Liu et al. proposed a novel second-order sliding mode control strategy based on Hermite neural networks for nonlinear vector control of synchronous reluctance motor drive systems. The effectiveness and superiority of this control strategy in handling external disturbances and parameter uncertainties were validated through comparative hardware-in-the-loop testing [30]. In [31], a decentralized event-triggered fault-tolerant echo-state network (ESN) direct adaptive control method is proposed for uncertain interconnected systems with input saturation, actuator faults, external disturbances, and unavailable states. A fuzzy inference system is used to estimate the control error and derive the adaptation laws, while the ESNs approximate ideal control laws and robust terms are introduced to enhance the stability of the closed-loop system. At the same time, Lin’s team has developed an intelligent servo drive system for permanent magnet-assisted synchronous reluctance motors, utilizing a recurrent wavelet fuzzy neural network and intelligent backstepping control to effectively handle the motor’s nonlinearity and time-varying characteristics [32]. Like References [26,33] introduces a control strategy that combines a nonlinear disturbance observer with dynamic surface control, addressing the issue of dimension explosion in traditional designs. The use of a RBFNN effectively approximates unknown system functions, enhancing both robustness and overall performance. In [34], this report introduces a novel I-PID-type controller for torque-driven flexible joint robots with input constraints, utilizing a double-loop cascade configuration and nonlinear control strategies to ensure global asymptotic stability despite disturbances and uncertainties. Real-time experiments on a two-degrees-of-freedom manipulator demonstrate the controller’s superior performance, confirmed through Lyapunov theory and the Barbashin–Krasovskii theorem. Bounemeur and Chemachema proposed a finite-time fault-tolerant adaptive fuzzy control method for uncertain interconnected systems, addressing input saturation, state-dependent actuator faults, external disturbances, and unmeasurable states. The method approximates the unknown ideal control laws using fuzzy systems, ensuring the stability of the closed-loop system, and the simulation results validate its effectiveness [35].

Furthermore, Long Short-Term Memory (LSTM), a subtype of Recurrent Neural Networks (RNNs), is distinguished by its ability to capture long-term dependencies in data, addressing the problem of vanishing gradients common in traditional RNNs. This capability is widely recognized in studies such as [36,37], emphasizing its utility in sequence modeling and temporal dependency challenges. Consequently, LSTMs are frequently utilized in applications like trajectory planning for autonomous vehicles and predicting system states, as noted in [37,38]. Addressing complex system dynamics and the limitations of traditional static detection methods, Reference [37] utilizes LSTM networks to detect data anomalies with temporal features. The superiority of LSTM networks is further demonstrated through an extensive evaluation tailored to specific application scenarios. Reference [39] demonstrates how LSTM networks, leveraging deep learning, effectively simulate the storage effect in snow-affected watersheds to enhance the accuracy of rainfall-runoff models. This application is underscored through a detailed case study, highlighting LSTM’s utility in hydrological modeling. Reference [40] describes the development of a deep learning object detector to determine the six degrees of freedom (6-DoF) between a UAV-mounted monocular camera and a drogue cone, enhancing the UAV’s spatial awareness and positioning for critical tasks like aerial refueling or docking maneuvers. This technology provides essential data for autonomous UAV operations, reducing reliance on manual control. The object detector’s performance is rigorously evaluated against the VICON motion tracking system, validating its accuracy and reliability for navigation and spatial tracking in practical applications.

Building on previous studies, this paper introduces an innovative approach for accurate tracking control of flexible single-joint robotic arms, effectively handling external disturbances and uncertainties through the use of adaptive neural network dynamic surface control techniques. Furthermore, it employs LSTM networks to predict and analyze vital system state variables. The principal contributions of this research are outlined as follows:

1. Unlike conventional approaches that separately employ neural networks or dynamic surface control, our strategy integrates these components into a cohesive framework. This integration enhances the adaptability and precision of the control system under uncertain conditions.
2. We introduce a novel application of nonlinear damping terms within the control law to effectively mitigate the impact of external disturbances, which is not commonly addressed in existing models.
3. Additionally, our method stands out by implementing an adaptive law that updates both neural network weights and system parameters in real-time, improving the system’s responsiveness to dynamic changes and uncertainties. We incorporate Long Short-Term Memory (LSTM) networks to predict and analyze system state data, a feature rarely utilized in traditional control systems for flexible joint manipulators. This predictive capability allows for preemptive adjustments, enhancing the robustness and effectiveness of the control strategy.

The structure of this paper is as follows: Sect 2 introduces the model transformation and problem description, detailing the conversion process and providing an overview of the problem. Sect 3 presents the control algorithm, which is based on the dynamic surface approach using neural networks. Sect 4 showcases numerical simulations conducted in MATLAB/Simulink to demonstrate the robustness and interference resilience of the proposed method. Finally, Sect 5 concludes with a summary of the main findings and suggestions for future research and development in this area.

## 2 Model transformation and problem description

### 2.1 Dynamic model of single-link flexible manipulator

**Fig 1:**
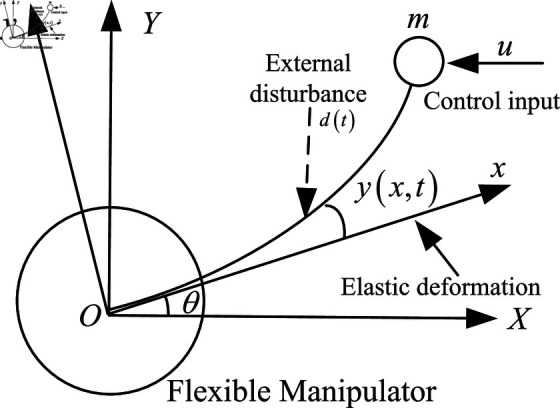
The response diagram of system state *x*(*t*) under constant disturbance.

The research focuses on a horizontally operating single-link flexible manipulator, comprising a connecting rod, joint, end effector, sensor, and control system, which collectively enable the execution of complex tasks [41]. As shown in Fig 1, the manipulator receives a constrained input signal *u*(*t*) and encounters external disturbances *d*(*t*) at the end effector.

To support controller design, we employ a standard dynamic model of a single-link flexible joint robot, serving as the foundation for subsequent controller development and analysis.


 {Iq¨+K (q-qm)+Mglsin ⁡ q=0Jq¨m-K (q-qm)=u. (1)

where q and $q_{m}$ denote the angular positions of the mechanical linkage and the rotor, respectively. I and J are the moments of inertia for the linkage and rotor, respectively, while K represents the joint stiffness coefficient. M, g, and l signify the mass of the linkage, gravitational acceleration, and the distance from the joint to the center of mass of the linkage, respectively. Lastly, u indicates the torque applied by the motor as an input.

By selecting the state variables as follows: $x_{1}=q$, $x_{2}=\dot{q}$, $x_{3}=q_{m}$, and $x_{4}={\dot{q}}_{m}$, and taking into account the influence of an external disturbance torque, we can express Eq (1) as follows:


$$\begin{array}{c} \{\begin{array}{c} {ẋ}_{1}=x_{2}\\ {ẋ}_{2}=a_{1}x_{3}+f_{1}\left(x_{1}\right)+\Delta_{1}(t)\\ {ẋ}_{3}=x_{4}\\ {ẋ}_{4}=a_{2}u+f_{2}\left(x_{1},x_{3}\right)+\Delta_{2}(t). \end{array} \end{array}$$ (2)

where, $a_{1}=\frac{K}{I}$, $a_{2}=\frac{1}{J}$, f1 (x1)=-MglI sin ⁡ x1-KIx1. $f_{2}\left(x_{1},x_{3}\right)=\frac{K}{J}\left(x_{1}-x_{3}\right)$; $\Delta_{1}(t)$ and $\Delta_{2}(t)$ represent external disturbance torques, and for positive values $\delta_{1}$ and $\delta_{2}$, it holds that $\left|\Delta_{1}(t)\right|\leq \delta_{1}$ and $\left|\Delta_{2}(t)\right|\leq \delta_{2}$.

**Assumption 1**: The target angle $x_{1d}$ is bounded, and both its first and second derivatives are well-defined, adhering to the condition that for a positive constant *ξ*, the sum of the squares of $x_{1d}$, its first derivative ${ẋ}_{1d}$, and its second derivative ${ẍ}_{1d}$ does not exceed *ξ*. The system’s physical parameters, specifically $a_{1}$ and $a_{2}$, are not known; however, they are constrained within known positive limits, with $a_{im}\leq a_{i}\leq a_{iM}$ for *i* = 1 , 2. The functional forms of $f_{1}(x_{1})$ and $f_{2}(x_{1},x_{3})$ are specific yet undisclosed.

### 2.2 Radial basis function neural network

**Fig 2:**
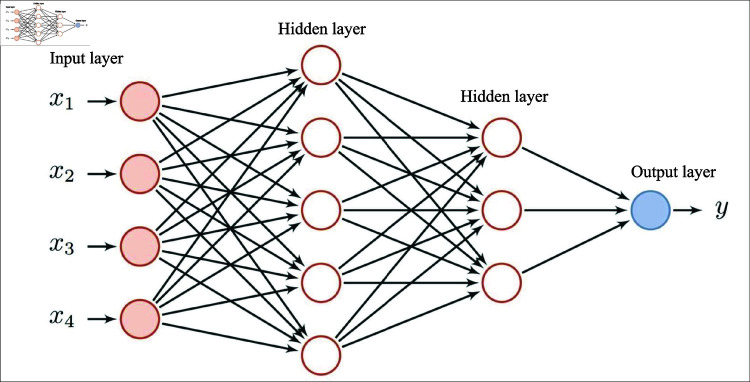
The schematic diagram of RBFNN structure.

**Remark 1**: The Radial Basis Function Neural Network (RBFNN) is a type of artificial neural network that uses radial basis functions, typically Gaussian functions, as activation functions in its hidden layer. It consists of three layers: an input layer, a hidden layer with radial basis functions, and an output layer (see Fig 2). RBFNNs are commonly used for function approximation, pattern recognition, and regression due to their ability to handle nonlinear problems. With a simple architecture and fewer model parameters, RBFNNs are easy to understand and interpret [42].

The RBFNN can accurately approximate any continuous nonlinear function. By utilizing an RBFNN to approximate the functions $f_{1}(x_{1})$ and $f_{2}(x_{1},x_{3})$, an optimal weight vector $\theta^{*}\in {\mathbb{R}}^{N}$ exists. With $\theta^{*}$, the neural network’s output $\theta^{*T}h(x)$ closely approximates *f *(*x*), with an approximation error $\sigma_{M}$ such that $\left|\sigma_{M}\right|$ is the maximum allowable error [43].


$$\begin{array}{c} f(x)=\theta^{*T}h(x)+\varepsilon^{*}. \end{array}$$ (3)

where, $\varepsilon^{*}$ denotes the approximation error, which is constrained to an absolute value no greater than the specified threshold $\varepsilon_{M}$. Furthermore, the function $h(x)\in {\mathbb{R}}^{N}$ represents the Gaussian basis functions, characterized by the following properties [43,44]:


$$\begin{array}{c} h_{i}(x)=\exp\left(-\frac{{∥x-d_{i}∥}^{2}}{2b^{2}}\right). \end{array}$$ (4)

where, $d_{i}\in {\mathbb{R}}^{n}$, where *i* = 1 , ⋯ , *N*, represents the center of the *i*-th Gaussian basis function, and *b* > 0 denotes the width of the Gaussian basis function. These centers, $d_{i}$, determine the positions in the input space around which the Gaussian basis functions are centered, and *b* controls the spread or width of these functions.

Since $\theta^{*}$ is unknown a priori, it is crucial to devise an adaptive control law to estimate it. Note that the elements of $\theta^{*}$ are bounded, assumed to be within known positive constants $\theta_{M}$, implying that the norm of $\theta^{*}$, $∥\theta^{*}∥$, does not exceed $\theta_{M}$. This suggests that the magnitude of each element of $\theta^{*}$ is capped at $\theta_{M}$.

### 2.3 Long short-term memory network

**Fig 3:**
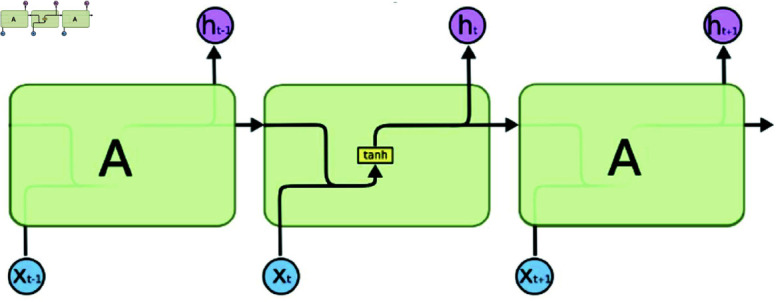
The schematic diagram of RNN structure.

**Remark 2**: The RNN is a neural network designed for sequential data, distinguished from traditional feedforward networks by its cyclic connections, which allow it to maintain a memory state during sequence processing (see Fig 3). A key variant of the RNN is the LSTM network, which excels at handling long-term dependencies in sequential data and addresses the vanishing gradient problem common in standard RNNs.

**Fig 4:**
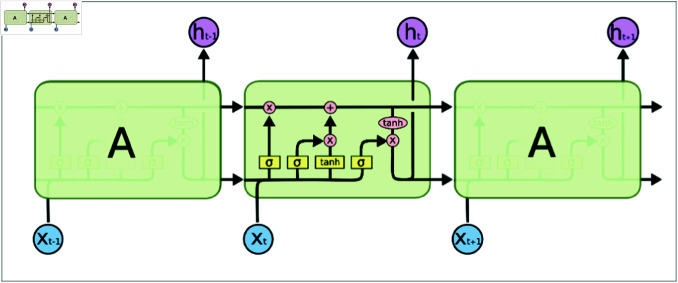
The schematic diagram of LSTM structure.

LSTM features a memory cell critical for storing and accessing information, controlled through gates including the input, forget, and output gates. The architecture of an LSTM is illustrated in the schematic in Fig 4.

In an LSTM network, the extent to which previous cell state information is forgotten is determined by the forget gate. This gate computes its value based on the current input and the previous time step’s hidden state, which are processed through a fully connected layer and a sigmoid function. The output ranges from 0 (complete forgetting) to 1 (full retention of previous state information). The formula for the forget gate is as


$$\begin{array}{c} f_{t}=\sigma \left(W_{f}\cdot \left[h_{t-1},x_{t}\right]+b_{f}\right). \end{array}$$ (5)

where, $W_{f}$ is the weight matrix, $b_{f}$ is the bias term, $h_{t-1}$ is the previous time step’s hidden state, $x_{t}$ is the current input, and *σ* is the sigmoid function.

In an LSTM network, it is crucial to decide what new information to store in the memory cell. This decision is governed by the input gate, which determines the segments of the memory cell to be updated, and a hyperbolic tangent (tanh) layer that generates a new candidate value vector for possible inclusion in the cell. The input gate and the candidate value vector are derived from the current input and the hidden state from the previous time step. The formula for the input gate is given as follows:


$$\begin{array}{l} i_{t}=\sigma \left(W_{i}\cdot \left[h_{t-1},x_{t}\right]+b_{i}\right)\;. \end{array}$$ (6)

$$\begin{array}{c} {\tilde{c}}_{t}=\tanh\left(W_{c}\cdot \left[h_{t-1},x_{t}\right]+b_{c}\right)\;. \end{array}$$ (7)

where $W_{C}$ and $W_{i}$ represent the weight matrices, and $b_{C}$ and $b_{i}$ are the associated bias terms. $h_{t-1}$ denotes the hidden state from the previous time step, and $x_{t}$ refers to the current input. The symbol *σ* denotes the sigmoid function, and tanh refers to the hyperbolic tangent function.

The memory cell in an LSTM network is updated by applying the decisions from the forget gate and the input gate. The cell state is multiplied by the forget gate’s value to discard certain state information, followed by adding the product of the input gate’s value and the candidate value, which introduces new state information. The formula for updating the cell state is as follows:


$$\begin{array}{c} c_{t}=f_{t}\times c_{t-1}+i_{t}\times {\tilde{c}}_{t}. \end{array}$$ (8)

where, $f_{t}$ represents the output of the forget gate, while $c_{t-1}$ corresponds to the previous time step’s cell state. $i_{t}$ denotes the input gate value, and ${\tilde{c}}_{t}$ is the candidate value.

The output is derived from the cell state, merging the current input with the hidden state from the previous time step via a fully connected layer. This layer applies a sigmoid function to set the output gate’s value, which ranges from 0 (no output) to 1 (full output). Subsequently, the cell state undergoes processing by a hyperbolic tangent (tanh) function, scaling it to a range of -1 to 1. This scaled value is then multiplied by the output gate’s value to compute the final hidden state. The operation of the output gate is described as follows:


$$\begin{array}{l} o_{t}=\sigma \left(W_{o}\cdot \left[h_{t-1},x_{t}\right]+b_{o}\right). \end{array}$$ (9)

$$\begin{array}{c} h_{t}=o_{t}\times \tanh\left(c_{t}\right). \end{array}$$ (10)

where, $W_{o}$ represents the weight matrix, $b_{o}$ corresponds to the bias term, and $h_{t-1}$ denotes the hidden state from the previous time step

### 2.4 Control objectives

**Fig 5:**
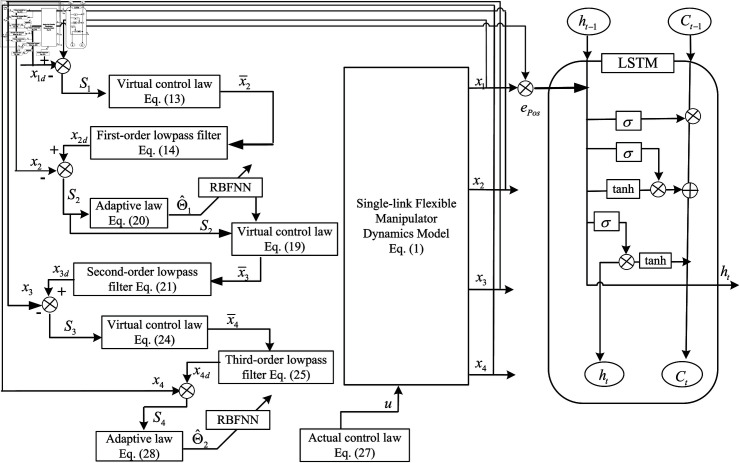
The schematic diagram of control logic.

The primary objective of this paper is to develop a control strategy that enables a flexible joint manipulator to accurately follow a predefined trajectory, specifically x1d= sin ⁡ t, for the angular position *θ* of its links. To achieve this goal, we propose an ADSC approach, which is enhanced by the integration of a RBFNN. The RBFNN is employed to facilitate real-time adjustments of the neural network weights and to identify unknown model parameters dynamically. Additionally, we incorporate LSTM networks to predict and analyze the system’s state variables, thereby improving the overall performance and responsiveness of the control system. A schematic representation of the proposed control strategy is illustrated in Fig 5.

## 3 Robust control of dynamic surface of adaptive RBF neural network

### 3.1 An overview of integrated control strategy

In this subsection, we provide a detailed introduction to the comprehensive control strategy used in this manuscript, as depicted in Fig 5. This strategy includes virtual control laws, multi-level low-pass filters, adaptive laws, RBFNN, and LSTM. These components work together to precisely adjust the performance of the single-link flexible manipulator. Through this approach, we have designed an efficient control architecture that enhances the system’s stability and responsiveness, ensuring optimal control performance under various operating conditions.

### 3.2 Robust controller design of single-link flexible manipulator

**Remark 3**: Dynamic Surface Control (DSC) is a control strategy designed to improve stability and tracking in nonlinear systems, especially under uncertainty and external disturbances. Commonly used in mechanical systems and robotics, DSC enhances performance by introducing dynamic feedback terms for asymptotic stability and improved trajectory tracking. Unlike backstepping control, DSC simplifies the design by using a “dynamic surface” to convert high-order derivatives into first-order, reducing system complexity and stabilizing oscillations and instabilities.

Following the “progressive” design method of inversion control, the robust controller’s design is divided into four steps.

Define the first dynamic surface error:


$$\begin{array}{c} S_{1}=x_{1}-x_{1d}. \end{array}$$ (11)

Taking the derivative, we get:


$$\begin{array}{c} {Ṡ}_{1}=x_{2}-{ẋ}_{1d}. \end{array}$$ (12)

Then, choose a virtual control as:


$$\begin{array}{c} {\bar{x}}_{2}=-c_{1}S_{1}+{ẋ}_{1d}. \end{array}$$ (13)

Take ${\bar{x}}_{2}$, where $c_{1}$ is a positive constant, and pass it through a first-order low-pass filter with a time constant of $\tau_{2}$ to generate a new state variable $x_{2d}$.


$$\begin{array}{c} \tau_{2}{ẋ}_{2d}+x_{2d}={\bar{x}}_{2},\;x_{2d}(0)={\bar{x}}_{2}(0). \end{array}$$ (14)

Define the second dynamic surface error:


$$\begin{array}{c} S_{2}=x_{2}-x_{2d}. \end{array}$$ (15)

The derivation of Eq (15) can be obtained


$$\begin{array}{c} {Ṡ}_{2}=a_{1}\left[x_{3}+\frac{1}{a_{1}}f_{1}\left(x_{1}\right)+\frac{1}{a_{1}}\left(\Delta_{1}-{ẋ}_{2_{d}}\right)\right]. \end{array}$$ (16)

Since $a_{1}$ and $f_{1}(x_{1})$ are unknown, construct the first RBFNN to approximate the unknown function $1∕a_{1}\cdot f_{1}(x_{1})$


$$\begin{array}{c} \frac{1}{a}f_{1}\left(x_{1}\right)=\Theta_{1}^{*T}h_{1}\left(x_{1}\right)+\varepsilon_{1}^{*}. \end{array}$$ (17)

where, $\left|\varepsilon_{1}^{*}\right|\leq \varepsilon_{M},∥\Theta_{1}^{*}∥\leq \Theta_{M}$.

Define


$$\begin{array}{c} \Theta_{1}^{T}=\left[\begin{array}{cc} \Theta_{1}^{*T} & \frac{1}{a_{1}} \end{array}\right],\;\varphi_{1}=\left[\begin{array}{c} h_{1}\left(x_{1}\right)\\ \frac{\delta_{1}^{2}S_{2}}{2\varepsilon }-{ẋ}_{2d}+c_{2}S_{2} \end{array}\right]. \end{array}$$ (18)

Given that $c_{2}$ is a positive constant and *ε* is a very small positive value, we use $\left(\delta_{1}^{2}S_{2}\right)∕(2\varepsilon )$ as a nonlinear damping term to mitigate the effects of $\Delta_{1}(t)$. To facilitate this, we introduce a virtual control variable, ${\bar{x}}_{3}$, specifically designed to achieve a targeted objective or response.


$$\begin{array}{c} {\bar{x}}_{3}=-{\hat{\Theta }}_{1}^{T}\varphi_{1}. \end{array}$$ (19)

where, ${\hat{\Theta }}_{1}$ represents the estimate of $\Theta_{1}$. The design of the adaptive law is as follows:


$$\begin{array}{c} {\dot{\hat{\Theta }}}_{1}=\Gamma_{1}\varphi_{1}S_{2}-\Gamma_{1}\eta_{1}{\hat{\Theta }}_{1}. \end{array}$$ (20)

where $\Gamma_{1}$ is a positive definite symmetric matrix, and $\eta_{1}$ is a positive real number. A first-order low-pass filter with a time constant $\tau_{3}$ is applied to $\bar{x_{3}}$, creating a new state variable $x_{3d}$ as follows:


$$\begin{array}{c} \tau_{3}{ẋ}_{3d}+x_{3d}={\bar{x}}_{3},\;x_{3d}(0)={\bar{x}}_{3}(0). \end{array}$$ (21)

Define the third dynamic surface error:


$$\begin{array}{c} S_{3}=x_{3}-x_{3d}. \end{array}$$ (22)

Computing the time derivative along the trajectory of Eq (22), it can be shown that


$$\begin{array}{c} {Ṡ}_{3}=x_{4}-{ẋ}_{3d}. \end{array}$$ (23)

Next, we define the virtual control variable ${\bar{x}}_{4}$ as


$$\begin{array}{c} {\bar{x}}_{4}=-c_{3}S_{3}+{ẋ}_{3d}. \end{array}$$ (24)

where $c_{3}$ is a positive constant, a first-order low-pass filter with a time constant $\tau_{4}$ is applied to the variable ${\bar{x}}_{4}$. This filtering procedure creates a new state variable, $x_{4d}$, with the transformation executed as follows:


$$\begin{array}{c} \tau_{4}{ẋ}_{4d}+x_{4d}={\bar{x}}_{4},\;x_{4d}(0)={\bar{x}}_{4}(0). \end{array}$$ (25)

where, $\left|\varepsilon_{2}^{*}\right|\leq \varepsilon_{M}$, and $∥\Theta_{2}^{*}∥\leq \Theta_{M}$.

We define the vectors as:


$$\begin{array}{c} \Theta_{2}^{T}=\left[\begin{array}{cc} \Theta_{2}^{*T} & \frac{1}{a_{2}} \end{array}\right],\;\varphi_{2}=\left[\begin{array}{c} h_{2}\left(x_{1},x_{3}\right)\\ \frac{\delta_{2}^{2}S_{4}}{2\varepsilon }-{ẋ}_{4d}+c_{4}S_{4} \end{array}\right]. \end{array}$$ (26)

where $c_{4}$ is a positive constant and *ε* is a very small positive value. The term $\left(\delta_{2}^{2}S_{4}\right)∕(2\varepsilon )$ is used as a nonlinear damping term to mitigate external disturbances represented by $\Delta_{2}(t)$. The established control law is specified as


$$\begin{array}{c} u=-\Theta_{2}^{*T}\varphi_{2}. \end{array}$$ (27)

where ${\hat{\Theta }}_{2}$ is the estimate of $\Theta_{2}$.

The adaptive law is formulated in the following manner:


$$\begin{array}{c} {\dot{\hat{\Theta }}}_{2}=\Gamma_{2}\varphi_{2}S_{4}-\Gamma_{2}\eta_{2}{\hat{\Theta }}_{2}. \end{array}$$ (28)

where $\Gamma_{2}$ represents a positive definite symmetric matrix, and $\eta_{2}$ is a positive design parameter.

**Remark 4**: Dynamic Surface Control (DSC) is a trajectory-tracking technique that does not rely on an extensive system model, making it well-suited for complex systems where precise modeling is challenging. DSC prioritizes accurate trajectory tracking and system stability, essential for high-precision tasks like robotic path following and exact positioning. Unlike traditional backstepping control methods, which may increase the dimensionality of the state space due to nonlinear terms [45–47], DSC simplifies the controller design and reduces computational load. The method often employs first-order filters to smooth trajectory error signals, mitigating the impact of rapid signal fluctuations and improving stability and tracking performance.

### 3.3 Analysis and proof of closed-loop system stability

**Remark 5**: In this subsection, we propose an adaptive NNDSC strategy for a flexible-joint robotic manipulator with inherent uncertainties. An RBFNN is employed to approximate the unknown functions in the system model, while nonlinear damping terms are introduced to counteract external disturbance torques. Adaptive laws are designed for the real-time update of neural network weights and system parameters. Using the Lyapunov method, we show that all signals in the closed-loop system remain semi-globally and uniformly bounded. Additionally, it is demonstrated that by properly tuning the controller’s parameters, tracking errors can be minimized to negligible levels, ensuring the system achieves highly accurate tracking performance.

The virtual control error is defined as:


$$\begin{array}{c} y_{i}=x_{id}-{\bar{x}}_{i},\;i=2,3,4. \end{array}$$ (29)

From Eq (14), Eq (21), Eq (25) and Eq (29), we can derive:


$$\begin{array}{c} {ẋ}_{id}=-\frac{y_{i}}{\tau },\;i=2,3,4. \end{array}$$ (30)

Additionally, we define:


$$\begin{array}{c} {\tilde{\Theta }}_{i}={\hat{\Theta }}_{i}-\Theta_{i},\;i=1,2. \end{array}$$ (31)

Taking the derivatives of the respective errors, we have:


$$\begin{array}{c} {Ṡ}_{1}=S_{2}+y_{2}+{\bar{x}}_{2}-{ẋ}_{1d}=S_{2}+y_{2}-c_{1}S_{1}. \end{array}$$ (32)

$$\begin{array}{c} \begin{array}{ccc} {Ṡ}_{2} & =a_{1}\left[S_{3}+y_{3}+{\bar{x}}_{3}+\Theta_{1}^{*T}h_{1}\left(x_{1}\right)+\varepsilon_{1}^{*}+\frac{1}{a_{1}}\left(\Delta_{1}-{ẋ}_{2d}\right)\right] & \\ & =a_{1}\left[S_{3}+y_{3}-{\hat{\Theta }}_{1}^{T}\varphi_{1}+\Theta_{1}^{T}\varphi_{1}+\varepsilon_{1}^{*}+\frac{1}{a_{1}}\left(\Delta_{1}-\frac{\delta_{1}^{2}S_{2}}{2\varepsilon }-c_{2}S_{2}\right)\right]\\ & =a_{1}\left(S_{3}+y_{3}-{\tilde{\Theta }}_{1}^{T}\varphi_{1}+\varepsilon_{1}^{*}\right)+\left(\Delta_{1}-\frac{\delta_{1}^{2}S_{2}}{2\varepsilon }-c_{2}S_{2}\right). \end{array} \end{array}$$ (33)

where, $\Theta_{1}^{T}\varphi_{1}$ is given by:


$$\begin{array}{c} \begin{array}{ccc} \Theta_{1}^{T}\varphi_{1} & =\left[\begin{array}{cc} \theta_{1}^{*T} & \frac{1}{a_{1}} \end{array}\right]\left[\begin{array}{c} h_{1}\\ \frac{\delta_{1}^{2}S_{2}}{2\varepsilon }-{ẋ}_{2d}+c_{2}S_{2} \end{array}\right] & \\ & =\Theta_{1}^{*T}h_{1}+\frac{1}{a_{1}}\left(\frac{\delta_{1}^{2}S_{2}}{2\varepsilon }-{ẋ}_{2d}+c_{2}S_{2}\right). \end{array} \end{array}$$ (34)

which implies that


$$\begin{array}{c} \Theta_{1}^{*T}h_{1}=\Theta_{1}^{T}\varphi_{1}+\frac{1}{a_{1}}\left(-\frac{\delta_{1}^{2}S_{2}}{2\varepsilon }+{ẋ}_{2d}-c_{2}S_{2}\right). \end{array}$$ (35)

$$\begin{array}{c} {Ṡ}_{3}=S_{4}+y_{4}+{\bar{x}}_{4}-{ẋ}_{3d}=S_{4}+y_{4}-c_{3}S_{3}. \end{array}$$ (36)

$$\begin{array}{c} \begin{array}{ccc} {Ṡ}_{4} & =a_{2}\left[u+\Theta_{2}^{*T}h_{2}\left(x_{1},x_{3}\right)+\varepsilon_{2}^{*}+\frac{1}{a_{2}}\left(\Delta_{2}-{ẋ}_{4d}\right)\right] & \\ & =a_{2}\left[-{\hat{\Theta }}_{2}^{T}\varphi_{2}+\Theta_{2}^{T}\varphi_{2}+\varepsilon_{2}^{*}+\frac{1}{a_{2}}\left(\Delta_{2}-\frac{\delta_{2}^{2}S_{4}}{2\varepsilon }-c_{4}S_{4}\right)\right]\\ & =a_{2}\left(-{\tilde{\Theta }}_{2}^{T}\varphi_{2}+\varepsilon_{2}^{*}\right)+\left({\dot{\Delta }}_{2}-\frac{\delta_{2}^{2}S_{4}}{2\varepsilon }-c_{4}S_{4}\right). \end{array} \end{array}$$ (37)

where


$$\begin{array}{c} \Theta_{2}^{T}\varphi_{2}=\left[\begin{array}{cc} \Theta_{2}^{*T} & \frac{1}{a_{2}} \end{array}\right]\left[\begin{array}{c} h_{2}\\ \frac{\delta_{2}^{2}S_{4}}{2\varepsilon }-{ẋ}_{4d}+c_{4}S_{4} \end{array}\right]=\Theta_{2}^{*T}h_{2}+\frac{1}{a_{2}}\left(\frac{\delta_{2}^{2}S_{4}}{2\varepsilon }-{ẋ}_{4d}+c_{4}S_{4}\right). \end{array}$$ (38)

which implies that


$$\begin{array}{c} \Theta_{2}^{*T}h_{2}=\Theta_{2}^{T}\varphi 2+\frac{1}{a_{2}}\left(-\frac{\delta_{2}^{2}S_{4}}{2\varepsilon }+{ẋ}_{4d}-c_{4}S_{4}\right). \end{array}$$ (39)

By deriving the error of each virtual control term, we get


$$\begin{array}{c} {ẏ}_{2}={ẋ}_{2d}-{\dot{\bar{x}}}_{2}=-\frac{y_{2}}{\tau_{2}}+c_{1}{Ṡ}_{1}-{ẍ}_{1d}. \end{array}$$ (40)

$$\begin{array}{c} {ẏ}_{3}={ẋ}_{3d}-{\dot{\bar{x}}}_{3}=-\frac{y_{3}}{\tau_{3}}+{\dot{\hat{\Theta }}}_{1}^{T}\varphi_{1}+{\hat{\Theta }}_{1}^{T}{\dot{\varphi }}_{1}. \end{array}$$ (41)

$$\begin{array}{c} {ẏ}_{4}={ẋ}_{4d}-{\dot{\bar{x}}}_{4}=-\frac{y_{4}}{\tau_{4}}+c_{3}{Ṡ}_{3}-{ẍ}_{3d}. \end{array}$$ (42)

According to Eq (14), Eq (21), Eqs (29)–(33), Eqs (37)–(42), there is an upper bound function $B_{i},i=2,3,4$, which as


$$\begin{array}{c} {ẏ}_{2}\leq -\frac{y_{2}}{\tau_{2}}+B_{2}\left(S_{1},S_{2},y_{2},{ẍ}_{1d}\right). \end{array}$$ (43)

$$\begin{array}{c} {ẏ}_{3}\leq -\frac{y_{3}}{\tau_{3}}+B_{3}\left(S_{1},S_{2},S_{3},y_{2},y_{3},{\tilde{\Theta }}_{1},x_{1d},{ẋ}_{1d},{ẍ}_{1d}\right). \end{array}$$ (44)

$$\begin{array}{c} {ẏ}_{4}\leq -\frac{y_{4}}{\tau_{4}}+B_{4}\left(S_{1},\cdots \;,S_{4},y_{2},y_{3},y_{4},{\tilde{\Theta }}_{1},x_{1d},{ẋ}_{1d},{ẍ}_{1d}\right). \end{array}$$ (45)

Consider the following compact set:


$$\begin{array}{c} \Omega_{1}:=\left\{\left(x_{1d},{ẋ}_{1d},{ẍ}_{1d}\right):x_{1d}^{2}+{ẋ}_{1d}^{2}+{ẍ}_{1d}^{2}\leq \xi \right\}. \end{array}$$ (46)

where *p* is any positive number. It is worth noting that $\Omega_{1}\;\times \;\Omega_{2}$ is also a compact set, and the absolute values of $B_{i}$, where *i* = 2 , 3 , 4, have maximum values on $\Omega_{1}\times \Omega_{2}$, denoted as $M_{i}$.

Consider the Lyapunov function:


$$\begin{array}{c} V_{1}=\frac{1}{2}\overset{4}{\underset{i=1}{\sum }}S_{i}^{2}. \end{array}$$ (47)

$$\begin{array}{c} V_{2}=\frac{1}{2}\overset{4}{\underset{i=2}{\sum }}y_{i}^{2}. \end{array}$$ (48)

$$\begin{array}{c} V_{3}=\frac{1}{2}a_{1}{\tilde{\Theta }}_{1}^{T}\Gamma_{1}^{-1}{\tilde{\Theta }}_{1}+\frac{1}{2}a_{2}{\tilde{\Theta }}_{2}^{T}\Gamma_{2}^{-1}{\tilde{\Theta }}_{2}. \end{array}$$ (49)

**Theorem 1**: Considering the closed-loop system, which incorporates the plant dynamics as defined in Eq (2) and the designated controller detailed in Eq (27), if Assumption 1 is met and the initial conditions ensure *V* ( 0 ) ≤ *p*, then it is possible to select tuning parameters $c_{i}$ for *i* = 1 , … , 4, $\tau_{i}$ for *i* = 2 , 3 , 4, *ε*, $\eta_{1}$, $\eta_{2}$, $\Gamma_{1}$, and $\Gamma_{2}$. These parameters can be adjusted such that all signals within the closed-loop system remain semi-globally and uniformly bounded.

**Proof**: Taking derivatives of $V_{1},V_{2}$, and $V_{3}$, respectively:


$$\begin{array}{c} \begin{array}{ccc} {\dot{V}}_{1} & =S_{1}\left(S_{2}+y_{2}-c_{1}S_{1}\right)+S_{2}[\;a_{1}\left(S_{3}+y_{3}-{\tilde{\Theta }}_{1}^{T}\varphi_{1}+\varepsilon_{1}^{*}\right) & \\ & +\left(\Delta_{1}-\frac{\delta_{1}^{2}S_{2}}{2\varepsilon }-c_{2}S_{2}\right)]+S_{3}\left(S_{4}+y_{4}-c_{3}S_{3}\right)\\ & +S_{4}\left[a_{2}\left(-{\tilde{\Theta }}_{2}^{T}\varphi_{2}+\varepsilon_{2}^{*}\right)+\left(\Delta_{2}-\frac{\delta_{2}^{2}S_{4}}{2\varepsilon }-c_{4}S_{4}\right)\right]. \end{array} \end{array}$$ (50)

$$\begin{array}{c} {\dot{V}}_{2}\leq \overset{4}{\underset{i=2}{\sum }}y_{i}\left(-\frac{y_{i}}{\tau_{i}}+B_{i}\right). \end{array}$$ (51)

$$\begin{array}{c} {\dot{V}}_{3}=a_{1}{\tilde{\Theta }}_{1}^{T}\varphi_{1}S_{2}-a_{1}{\tilde{\Theta }}_{1}^{T}\eta_{1}{\hat{\Theta }}_{1}+a_{2}{\tilde{\Theta }}_{2}^{T}\varphi_{2}S_{4}-a_{2}{\tilde{\Theta }}_{2}^{T}\eta_{2}{\hat{\Theta }}_{2}. \end{array}$$ (52)

The inequalities $\left(\delta_{1}^{2}S_{2}^{2}\right)∕(2\varepsilon )+\varepsilon ∕2\geq \left|\delta_{1}\right|\left|S_{2}\right|\geq \Delta_{1}S_{2}$ and $\left(\delta_{2}^{2}S_{4}^{2}\right)∕(2\varepsilon )+\varepsilon ∕2\geq \left|\delta_{2}\right|\left|S_{4}\right|\geq \Delta_{2}S_{4}$ are of significant importance. These inequalities, which link various parameters in the context of a specific problem, offer valuable insights into the interplay of different variables.

**Remark 6**: The first set of inequalities illustrates the relationship between the squared deviations $\delta_{1}^{2}$ and $S_{2}^{2}$ relative to the energy scale *ε*, as well as their influence on the absolute values of $\delta_{1}$ and $S_{2}$. These inequalities also highlight the connection between the product of $\left|\delta_{1}\right|$ and $\left|S_{2}\right|$ and the product of $\Delta_{1}$ and $S_{2}$, revealing their roles in the system’s dynamics. Similarly, the second set of inequalities involving $\delta_{2}$ and $S_{4}$ follows the same pattern, providing insights into their interrelations and constraints in the context of the problem.

**Remark 7**: In this paper, we apply Lyapunov’s theory to evaluate the stability of the closed-loop system. It is crucial to highlight that Lyapunov’s theory is primarily used to analyze the stability of equilibrium points in a system. As such, we have rigorously established the existence of at least one equilibrium point within the closed-loop control system and have employed a Lyapunov function based on this equilibrium point to demonstrate the uniform boundedness and stability of the system’s states. The closed-loop control strategy we propose guarantees that the system can reach and maintain this equilibrium state under specific conditions, thereby achieving the desired control objectives.

From Eq (50), Eq (51) and Eq (53), we can obtain


$$\begin{array}{c} \begin{array}{ccc} \dot{V}\leq & \left|S_{1}\right|\left|S_{2}\right|+\left|S_{1}\right|\left|y_{2}\right|+a_{1}\left|S_{2}\right|\left|S_{3}\right|+a_{1}\left|S_{2}\right|\left|y_{3}\right|+\left|S_{3}\right|\left|S_{4}\right|+\left|S_{3}\right|\left|y_{4}\right| & \\ & -\overset{4}{\underset{i=1}{\sum }}c_{i}S_{i}^{2}+a_{1}\left|S_{2}\right|\left|\varepsilon_{1}^{*}\right|+a_{2}\left|S_{4}\right|\left|\varepsilon_{2}^{*}\right|+\varepsilon +\overset{4}{\underset{i=2}{\sum }}\left(-\frac{y_{i}^{2}}{\tau_{i}}+\left|B_{i}\right|\left|y_{i}\right|\right)\\ & -a_{1}{\tilde{\Theta }}_{1}^{T}\eta_{1}{\hat{\Theta }}_{1}-a_{2}{\tilde{\Theta }}_{2}^{T}\eta_{2}{\hat{\Theta }}_{2}. \end{array} \end{array}$$ (53)

By utilizing the Young’s inequality and the inequality $2{\tilde{\Theta }}^{T}\hat{\Theta }\geq |\tilde{\Theta }{|}^{2}-|\Theta {|}^{2}$, we can derive the following expression for $\dot{V}$:


$$\begin{array}{c} \begin{array}{ccc} \dot{V}\leq & \frac{1}{2}\left(S_{1}^{2}+S_{2}^{2}\right)+\frac{1}{2}\left(S_{1}^{2}+y_{2}^{2}\right)+\frac{a_{1}}{2}\left(S_{2}^{2}+S_{3}^{2}\right) & \\ & +\frac{a_{1}}{2}\left(S_{2}^{2}+y_{3}^{2}\right)+\frac{1}{2}\left(S_{3}^{2}+S_{4}^{2}\right)+\frac{1}{2}\left(S_{3}^{2}+y_{4}^{2}\right)\\ & -\overset{4}{\underset{i=1}{\sum }}c_{i}S_{i}^{2}+\frac{a_{1}}{2}\left(S_{2}^{2}+\varepsilon_{1}^{*2}\right)+\frac{a_{2}}{2}\left(S_{4}^{2}+\varepsilon_{2}^{*}\right)\\ & +\varepsilon +\overset{4}{\underset{i=2}{\sum }}\left(-\frac{y_{i}^{2}}{\tau_{i}}+\frac{B_{i}^{2}y_{i}^{2}}{2\varepsilon }+\frac{\varepsilon }{2}\right)\\ & -\frac{\eta_{1}a_{1}}{2}\left({∥{\tilde{\Theta }}_{1}∥}^{2}-{∥\Theta_{1}∥}^{2}\right)-\frac{\eta_{2}a_{2}}{2}\left({∥{\tilde{\Theta }}_{2}∥}^{2}-{∥\Theta_{2}∥}^{2}\right). \end{array} \end{array}$$ (54)

This expression represents an upper bound on the time derivative of a certain function *V *. It appears to be a complex combination of various terms and variables, including $S_{i}$, $y_{i}$, $a_{i}$, $\varepsilon_{i}^{*}$, $c_{i}$, $\tau_{i}$, $B_{i}$, and $\eta_{i}$, as well as the norms of ${\tilde{\Theta }}_{1}$, $\Theta_{1}$, ${\tilde{\Theta }}_{2}$, and $\Theta_{2}$. The specific context or application of this inequality would be needed to provide further interpretation or analysis.

Certainly, let’s further simplify and organize the expression for $\dot{V}$:


$$\begin{array}{c} \begin{array}{ccc} \dot{V}\leq & \left(1-c_{1}\right)S_{1}^{2}+\left[\left(\frac{1}{2}+\frac{3a_{1}}{2}-c_{2}\right)S_{2}^{2}+\left(1+\frac{a_{1}}{2}-c_{3}\right)\right]S_{3}^{2}+ & \\ & \left(\frac{1}{2}+\frac{a_{2}}{2}-c_{4}\right)S_{4}^{2}+\left(\frac{1}{2}+\frac{B_{2}^{2}}{2\varepsilon }-\frac{1}{\tau_{2}}\right)y_{2}^{2}+\\ & \left(\frac{a_{1}}{2}+\frac{B_{3}^{2}}{2\varepsilon }-\frac{1}{\tau_{3}}\right)y_{3}^{2}+\left(\frac{1}{2}+\frac{B_{4}^{2}}{2\varepsilon }-\frac{1}{\tau_{4}}\right)y_{4}^{2}-\\ & \frac{\eta_{1}a_{1}}{2\lambda_{\max}\left(\Gamma_{1}^{-1}\right)}{\tilde{\Theta }}_{1}^{T}\Gamma_{1}^{-1}{\tilde{\Theta }}_{1}-\frac{\eta_{2}a_{2}}{2\lambda_{\max}\left(\Gamma_{2}^{-1}\right)}{\tilde{\Theta }}_{2}^{T}\Gamma_{2}^{-1}{\tilde{\Theta }}_{2}+\\ & \frac{5}{2}\varepsilon +\frac{\eta_{1}a_{1}}{2}{∥\Theta_{1}∥}^{2}+\frac{\eta_{2}a_{2}}{2}{∥\Theta_{2}∥}^{2}+\frac{a_{1}}{2}\varepsilon_{1}^{*2}+\frac{a_{2}}{2}\varepsilon_{2}^{*2}. \end{array} \end{array}$$ (55)

The specified inequality and conditions concerning $\eta_{0}$, $\eta_{1}$, and $\eta_{2}$ indicate that these parameter selections enable the establishment of an upper bound for the left-hand side expression, which is defined as $-2rV_{3}\eta_{0}$. Here, $\lambda_{\text{max}}(\cdot )$ denotes the maximum eigenvalue of the specified matrix. Given these conditions:


$$0<\eta_{0}\leq 1.0,\eta_{1}\geq 2r\lambda_{\text{max }}\left(\Gamma_{1}^{-1}\right)\eta_{0},\eta_{2}\geq 2r\lambda_{\text{max }}\left(\Gamma_{2}^{-1}\right)\eta_{0}.$$

The significance of this result lies in its ability to demonstrate that the expression on the left-hand side of the inequality can be constrained within the bound of $-2rV_{3}\eta_{0}$. In control theory and stability analysis, this finding offers a valuable method for regulating and restricting the system’s behavior. The specific values of $\eta_{0}$, $\eta_{1}$, and $\eta_{2}$ along with the properties of the matrices $\Gamma_{1}^{-1}$ and $\Gamma_{2}^{-1}$ will determine the exact control and stability characteristics of the system.

The control parameters are determined and configured as follows:


$$\begin{array}{c} \begin{array}{ccc} & c_{1}\geq 1+r,c_{2}\geq \frac{1}{2}+\frac{3a_{1M}}{2}+r & \\ & c_{3}\geq 1+\frac{a_{1M}}{2}+r,c_{4}\geq \frac{1}{2}+\frac{a_{2M}}{2}+r\\ & \frac{1}{\tau_{2}}\geq \frac{1}{2}+\frac{M_{2}^{2}}{2\varepsilon }+r,\frac{1}{\tau_{3}}\geq \frac{a_{1M}}{2}+\frac{M_{3}^{2}}{2\varepsilon }+r,\frac{1}{\tau_{4}}\geq \frac{1}{2}+\frac{M_{4}^{2}}{2\varepsilon }+r\\ & \eta_{1}\geq 2r\lambda_{\max}\left(\Gamma_{1}^{-1}\right)\eta_{0},\eta_{2}\geq 2r\lambda_{\max}\left(\Gamma_{2}^{-1}\right)\eta_{0}. \end{array} \end{array}$$ (56)

where the parameter *r* is a positive number that needs to be determined or designed.

Then


$$\begin{array}{c} \begin{array}{ccc} \dot{V}\leq & -2r\left(V_{1}+V_{2}+\eta_{0}V_{3}\right)+\frac{5}{2}\varepsilon +\frac{\eta_{1}a_{1}}{2}{∥\Theta_{1}∥}^{2}+ & \\ & \frac{\eta_{2}a_{2}}{2}{∥\Theta_{2}∥}^{2}+\frac{a_{1}}{2}\varepsilon_{1}^{{}^{2}}+\frac{a_{2}}{2}\varepsilon_{2}^{2}+\overset{4}{\underset{i=2}{\sum }}\left(\frac{M_{i}^{2}}{2\varepsilon }\frac{B_{i}^{2}}{M_{i}^{2}}-\frac{M_{i}^{2}}{2\varepsilon }\right)y_{i}^{2}. \end{array} \end{array}$$ (57)

Based on Assumption 1 and the conditions $\left|\varepsilon_{i}^{*}\right|\leq \varepsilon_{M}$ and $∥\Theta_{i}^{*}∥\leq \Theta_{M}$ for *i* = 1 , 2, it can be inferred that the expression $\frac{5}{2}\varepsilon +\frac{\eta_{1}a_{1}}{2}{∥\Theta_{1}∥}^{2}+\frac{\eta_{2}a_{2}}{2}{∥\Theta_{2}∥}^{2}+\frac{a_{1}}{2}\varepsilon_{1}^{*2}+\frac{a_{2}}{2}\varepsilon_{2}^{*2}$ has a maximum value denoted as *Q*. Selecting *r* such that *r* ≥ *Q* ∕ ( 2*p* ) , we have the following


$$\begin{array}{c} \dot{V}\leq -2r\eta_{0}V+Q+\overset{4}{\underset{i=2}{\sum }}\left(\frac{B_{i}^{2}}{M_{i}^{2}}-1\right)\frac{M_{i}^{2}y_{i}^{2}}{2\varepsilon }. \end{array}$$ (58)

Given that the condition $\left|B_{i}\right|\leq M_{i}$ is met when *V* = *p*, it results in $\dot{V}\leq -2rp+Q\leq 0$ at *V* = *p*. Thus, *V* ≤ *p* forms an invariant set. This means that if *V* ( 0 ) ≤ *p*, then *V *(*t*) will also be  ≤ *p* for all *t* > 0. Assuming *V* ( 0 ) ≤ *p*, we find:


$$\begin{array}{c} \dot{V}\leq -2r\eta_{0}V+Q. \end{array}$$ (59)

Solving the inequality above, we obtain:


$$\begin{array}{c} V\leq \frac{Q}{2r\eta_{0}}+\left(V(0)-\frac{Q}{2r\eta_{0}}\right)e^{-2\eta_{0}t}. \end{array}$$ (60)

It is evident that all signals in the closed-loop system are semi-globally bounded. With $V=\frac{Q}{2r\eta_{0}}$, $\dot{V}$ equates to zero. Thus, as *t* approaches infinity, *V * converges to $\frac{Q}{2r\eta_{0}}$.

## 4 Simulation examples

To more effectively validate the proposed method in this paper, a nonlinear adaptive backstepping control utilizing an RBF neural network, referred to as NN-ABSC, is designed and evaluated through simulation. Given the agreement on the simulation object, specific Lyapunov functions are crafted for the NN-ABSC approach.


$$\begin{array}{c} V_{ABSC}=\frac{1}{2}\left(e_{1}^{2}+e_{2}^{2}+e_{3}^{2}+e_{4}^{2}\right)+\frac{1}{2\kappa_{1}}{\tilde{\Theta }}^{T}\tilde{\Theta }. \end{array}$$ (61)

where, $e_{1}$, $e_{2}$, $e_{3}$ and $e_{4}$ represent the error term. $\kappa_{1}$ is design coefficients, with the condittion that $\kappa_{1}>0$.

Let


$$\begin{array}{l} {\tilde{\varphi }}_{3}=\varphi_{3}-{\hat{\varphi }}_{3}. \end{array}$$ (62)

$$\begin{array}{c} \tilde{\Theta }=\hat{\Theta }-\Theta^{*}. \end{array}$$ (63)

The neural network architecture implemented in the NN-ABSC framework is configured as 2-5-1, with its input defned as $x=\left[\begin{array}{cc} x_{2} & x_{3} \end{array}\right]$.

The NN-ABSC is illustrated as a systematic approach, then have


$$\begin{array}{c} u_{ABSC}=\frac{1}{{\hat{\varphi }}_{3}}\left[-{\hat{f}}_{3}\left(x_{2},x_{3}\right)-\Delta_{2}+{ẋ}_{4d}-\eta \text{sgn}e_{4}-k_{3}e_{4}-e_{3}\right]. \end{array}$$ (64)

In this section, we conduct a numerical simulation using MATLAB/Simulink to evaluate the performance and effectiveness of the control algorithm developed for the flexible manipulator. To ensure a fair comparison between the two methods, it is essential that the neural networks in both approaches start with identical initial weight configurations, learning rates, network structures, and the same number of neurons in the hidden layers. These precautions help eliminate any performance discrepancies that might arise from differing training conditions, ensuring that the comparison reflects the true effectiveness of each method rather than being influenced by variations in the training setup.

In addition, the RBF neural network parameters of the two methods are the same. In the simulation, the primary control objective is to develop a control law for the link angle *q* in order to accurately track the desired trajectory, which is defined as x1d= sin ⁡ t. Assuming disturbance torques are given by $\Delta_{1}=0.1\sin(2t)$ and $\Delta_{2}=0.1\cos(2t)$. The physical parameters of the system are selected as: $I=J=1kg\cdot m^{2},Mgl=5N\cdot m,K=40N\cdot m∕rad$. These parameters are used solely for constructing the object in the simulation. As a result, the true values of $\delta_{1}=0.1$ and $\delta_{2}=0.1$, as well as $1∕a_{1}$ and $1∕a_{2}$, are respectively equal to 1∕40 and 1. The initial state of the system is set to $x(0)={\left[\begin{array}{cccc} 0.1 & 0 & 0 & 0 \end{array}\right]}^{T}$.

According to the definitions of ${\bar{x}}_{2}$, ${\bar{x}}_{3}$, and ${\bar{x}}_{4}$, we can obtain the initial values for the three filter equations Eq (14), Eq (21) and Eq (25). Since ${\bar{x}}_{2}(0)=-c_{1}S_{1}(0)+{ẋ}_{1d}(0)$, then $x_{2d}(0)={\bar{x}}_{2}(0)=-c_{1}(x_{1}(0)-x_{1d}(0))+{ẋ}_{1d}(0)=-5(0.1-0)+1=0.5$. Since the network weights are all initialized to 0, ${\bar{x}}_{3}(0)=-\hat{\theta }1^{T}(0)\varphi_{1}=0$. Therefore, according to Eq (21), we can obtain $x_{3d}(0)={\bar{x}}_{3}(0)=0$. As for ${\bar{x}}_{4}(0)=-c_{3}S_{3}(0)+{ẋ}_{3d}(0)=-c_{3}(x_{3}(0)-x_{3d}(0))+{ẋ}_{3d}(0)$, where ${ẋ}_{3d}(0)=({\bar{x}}_{3}(0)-x_{3d}(0))∕\tau_{3}=0$, then $x_{4d}(0)={\bar{x}}_{4}(0)=0$.

According to the expression for *V *, we have $V(0)=V_{1}(0)+V_{2}(0)+V_{3}(0)$. Since the initial values of the functions approximated by the RBFNN, $1∕a_{1}\cdot f_{1}\left(x_{1}\right)$ and $1∕a_{2}\cdot f_{2}\left(x_{1},x_{3}\right)$, are assumed to be zero, it might seem reasonable to also assume that the initial weights of the RBFNN could be set to zero. The weights used for approximation by the RBFNNs are all initialized to 0. Therefore, we can set $V_{3}(0)=0$. Thus, it can be determined that V(0)=0.13. Following the condition *p* ≥ *V* ( 0 ) , we design p=0.23. According to the equation *r* ≥ *Q* ∕ ( 2*p* ) , we design the value of *r*. Since $Q=\frac{5}{2}\varepsilon +\frac{\eta_{1}a_{1}}{2}{\left|\Theta_{1}\right|}^{2}+\frac{\eta_{2}a_{2}}{2}{\left|\Theta_{2}\right|}^{2}+\frac{a_{1}}{2}\varepsilon_{1}^{-2}+\frac{a_{2}}{2}\varepsilon_{2}^{-2}$, we can design very small values for $\eta_{1}$ and $\eta_{2}$ to minimize *Q*.

In the simulation, it was found that small values for $\eta_{1}$ and $\eta_{2}$ are required in the adaptive laws (20 and 28) to obtain satisfactory results. As a result, it is essential to select a very small value for $\eta_{0}$. The parameter *r* is chosen based on the condition *r* ≥ *Q* ∕ ( 2*p* ) . It is important to note that although *Q* is dependent on $\eta_{1}$ and $\eta_{2}$, both of these parameters are also influenced by the value of *r*. Additionally, the key parameters for both control methods are summarized in Table 1.

**Table 1:** The parameters for two different control methods.

Control method	Proposed control	NN-ABSC
Parameters	Controller for proposed method:	Controller for the NN-ABSC:
	$$\begin{array}{c} c_{1}=5,c_{2}=6,c_{3}=25,c_{4}=5\\ M_{2}=M_{3}=M_{4}=1.0\\ \text{RBFNN parameters:}\\ d_{1}=\left[\begin{array}{ccccccc} -3 & -2 & -1 & 0 & 1 & 2 & 3 \end{array}\right]\\ d_{2}=\left[\begin{array}{ccccccc} -1.5 & -1 & -0.5 & 0 & 0.5 & 1 & 1.5\\ -1.5 & -1 & -0.5 & 0 & 0.5 & 1 & 1.5 \end{array}\right]\\ b_{1}=b_{2}=10,\varepsilon =0.01\\ \tau_{2}=\tau_{3}=\tau_{4}=0.01\\ \Gamma_{1}=\mathrm{diag}[3,3,3,3,3,3,3,0.001]\\ \Gamma_{2}=\mathrm{diag}[10,10,10,10,10,10,10,0.4] \end{array}$$	$$\begin{array}{c} \kappa_{1}=1,k_{3}=0.2,\eta =0.1\\ \text{RBFNN parameters:}\\ \begin{array}{ccc} & \xi_{ABSC}= & \\ & \left[\begin{array}{ccccc} -1 & -0.5 & 0 & 0.5 & 1\\ -1 & -0.5 & 0 & 0.5 & 1 \end{array}\right] \end{array}\\ \Gamma_{ABSC}=\mathrm{diag}[3,3,3,3,0.001] \end{array}$$

**Fig 6:**
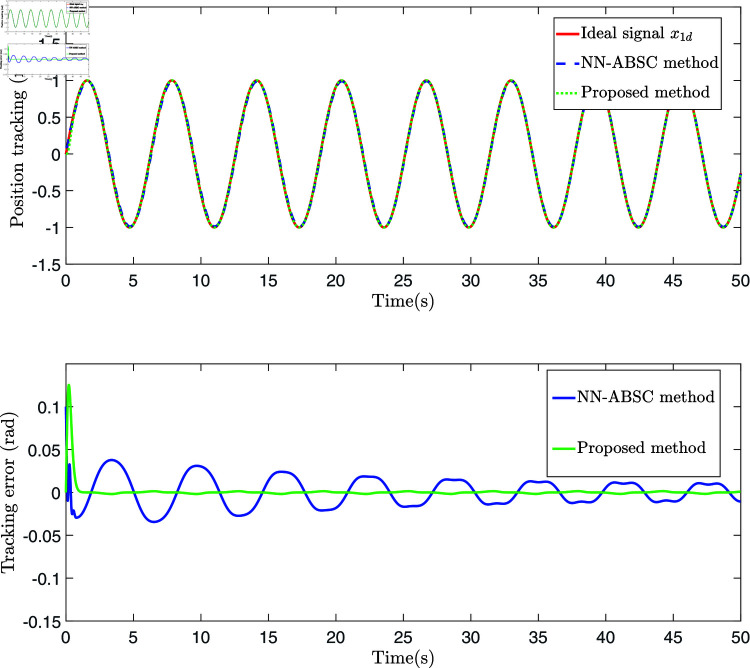
The position tracking and error of a single-link flexible manipulator.

**Fig 7:**
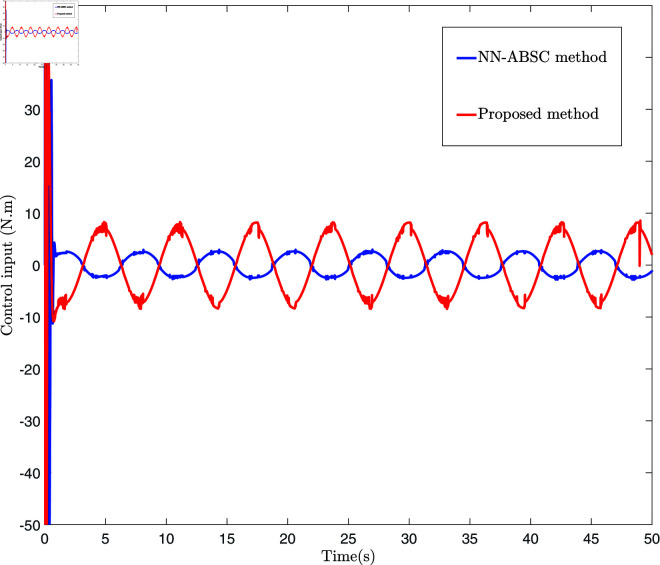
The schematic diagram of system control input.

**Fig 8:**
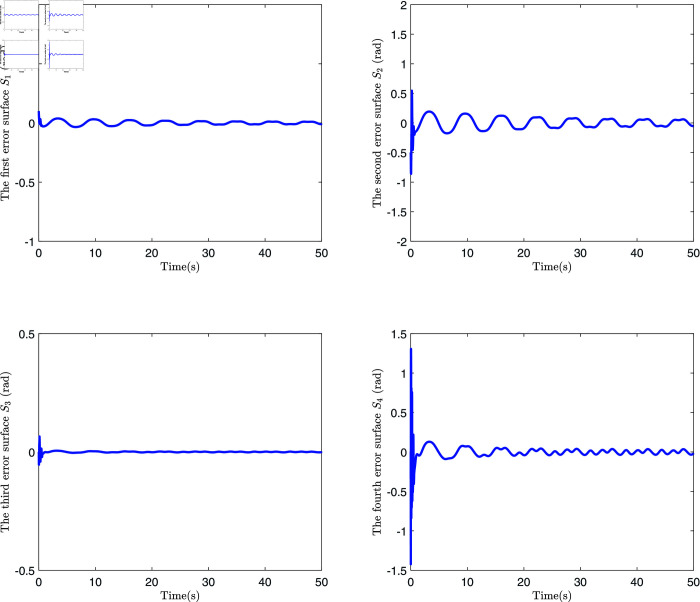
The schematic diagram of dynamic surface error.

**Fig 9:**
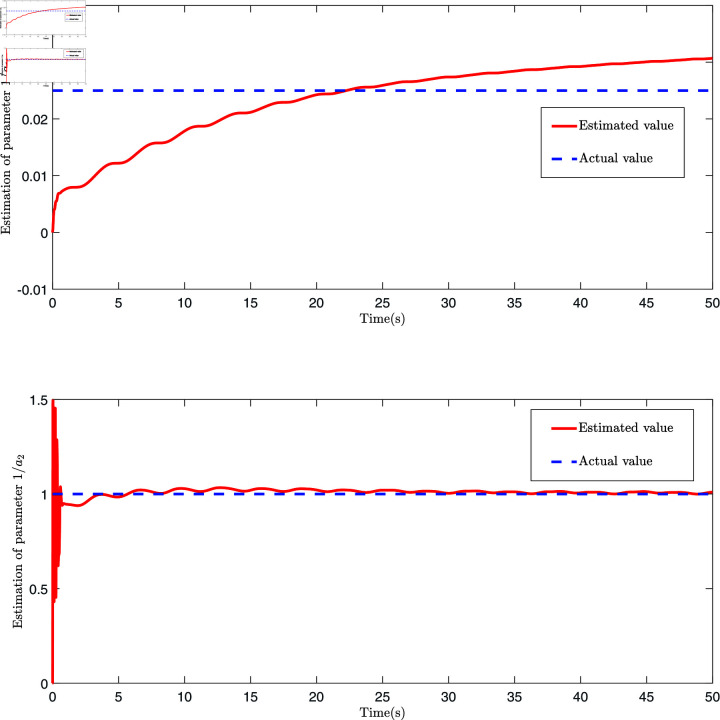
The schematic diagram of parameter $\text{1}/{\text{a}}_{\text{1}}$, $\text{1}/{\text{a}}_{\text{2}}$ and its estimation.

**Fig 10:**
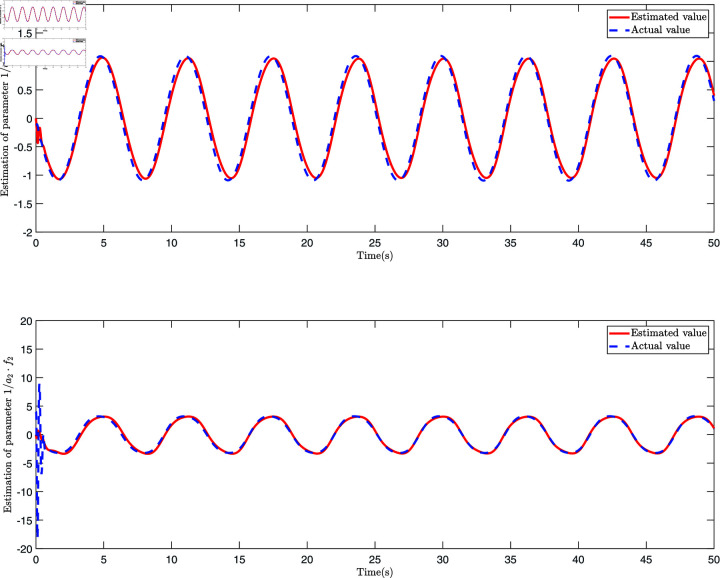
The schematic diagram of function $\text{1}/{\text{a}}_{\text{1}}\cdot {\text{f }}_{\text{1}}$, $\text{1}/{\text{a}}_{\text{2}}\cdot f_{2}$ and its estimation.

**Fig 11:**
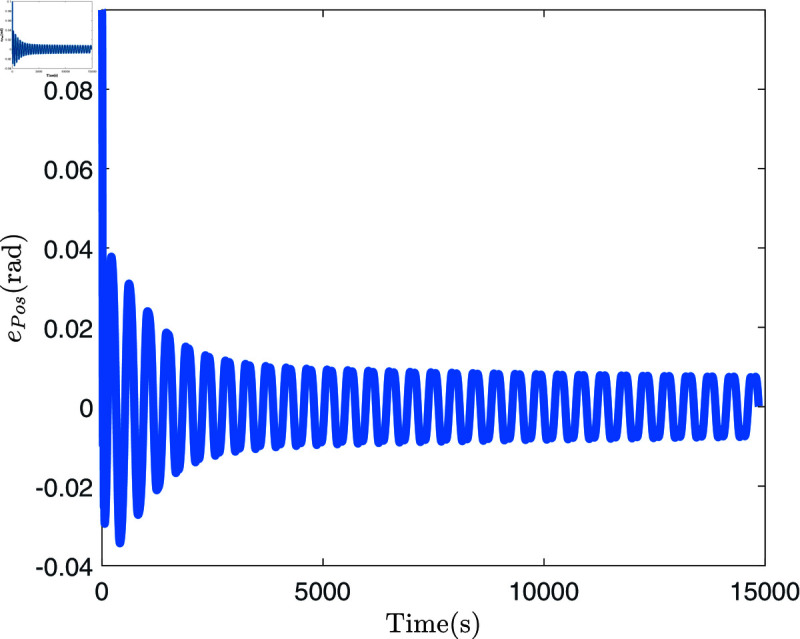
The diagram of position error.

**Fig 12:**
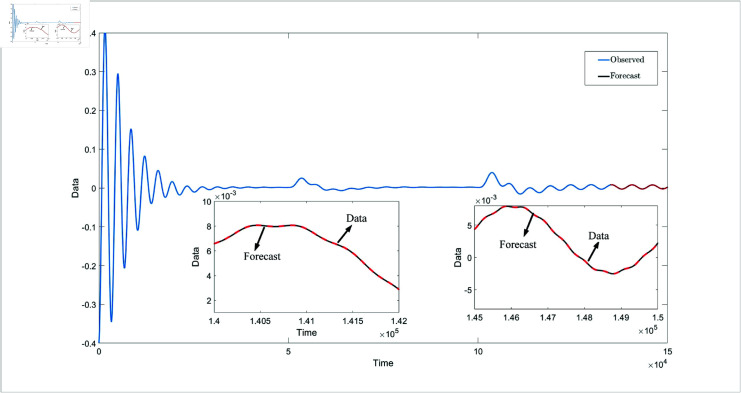
Prediction of position error.

The control law Eq (27), adaptive law Eq (20), and Eq (28) are employed for the simulation, and the results are presented in Figs 6, 7, 8, 9, 10, 11, and 12. Fig 6 presents three distinct types of signals: the ideal signal (depicted as a solid red line), the NN-ABSC method (shown as a blue dotted line), and the proposed method (illustrated with a green dotted line). The ideal signal illustrates the target trajectory for the system, while the other two lines indicate the performance of two different control methods in a practical setting. The lower segment of Fig 6 displays the tracking errors for both the NN-ABSC method (in blue) and the proposed method (in green). This figure highlights the superior accuracy and stability of the proposed control method in maintaining the trajectory of the single-link flexible manipulator in line with the ideal signal, showing significant enhancements over the NN-ABSC method. Fig 7 compares the control inputs for the same system, detailing the dynamics between control force and time for both the NN-ABSC method and the proposed method when managing a single-link flexible manipulator. It reveals that the proposed method is more efficient and precise in its application of control force, making it potentially more suited for scenarios requiring delicate operations.

Fig 8 presents four dynamic surface error curves designed in this paper, and it can be seen from Fig 8 that the system’s dynamic surface error ($S_{1}$, $S_{2}$, $S_{3}$ and $S_{4}$) converges to zero. The system parameters and their estimations are presented in Fig 9. As shown in this figure, the Radial Basis Function Neural Network (RBFNN) utilized in this study successfully estimates the unknown parameters. Similarly, Fig 10 illustrates the functions and their corresponding estimations. From this, it is clear that the RBFNN is effective in approximating the unknown functions. Fig 11 depicts the time variation of the system’s position error. The figure reveals a significant initial peak in the position error, which rapidly decreases and gradually stabilizes, showing a noticeable attenuation in oscillations. Fig 12 compares the observed and predicted position errors of the flexible manipulator. The blue curve represents the actual observed position error, while the red curve indicates the predicted position error. The inset provides a zoomed-in view of a specific time period, allowing a more detailed comparison of the observed data and predictions. From Figs 11 and 12, it is evident that by accurately predicting the future position error, the control system can proactively adjust its parameters to prevent large errors, which could otherwise lead to performance degradation or system instability. This predictive capability is particularly crucial for applications where safety is a primary concern.

## 5 Discussion and future work

This study proposes a robust control methodology, termed ADSC, for a single-joint flexible robotic manipulator, effectively addressing system uncertainties. The approach combines RBFNN for accurate approximation of unknown system functions, with the introduction of nonlinear damping terms to mitigate the impact of external disturbances. A novel adaptive law is established to continuously estimate the neural network weights and unknown model parameters, ensuring that the system remains adaptable under various operating conditions. Additionally, LSTM networks are employed to analyze and predict state variable position errors, enhancing the predictive capabilities of the system. Simulation results confirm the robustness and effectiveness of the proposed control approach, demonstrating its suitability for managing uncertainties and disturbances in real-time applications.

Furthermore, future research could explore the integration of more advanced deep learning techniques, such as reinforcement learning and convolutional neural networks, to further improve state analysis and prediction. Cross-disciplinary collaboration in fields such as control engineering, machine learning, robotics, and materials science is essential for developing deeper insights and innovative solutions to future challenges in flexible robotic systems.

## Supporting information

S1 Text Paper program. (PDF)
