# Supplementary material for: Adaptive control and state error prediction of flexible manipulators using radial basis function neural network and dynamic surface control method
Source: PLoS One. 2025 Feb 26;20(2):e0318601. doi: 10.1371/journal.pone.0318601 (PMC11864562; doi:10.1371/journal.pone.0318601)
Supplement: S1 Text — (PDF) [file pone.0318601.s001.pdf]

```
%%%%%%%%%%% Paper main program code
%%%%%%%%%%%%%%    ADSC CONTROL
function [sys,x0,str,ts]=s_function(t,x,u,flag)
switch flag,
case 0,
    [sys,x0,str,ts]=mdlInitializeSizes;
case 1,
    sys=mdlDerivatives(t,x,u);
case 3,
    sys=mdlOutputs(t,x,u);
case {2, 4, 9 }
    sys = [];
otherwise
    error(['Unhandled flag = ',num2str(flag)]);
end
function [sys,x0,str,ts]=mdlInitializeSizes
global hidden3 c3 b3;
sizes = simsizes;
sizes.NumContStates    = 10;
sizes.NumDiscStates    = 0;
sizes.NumOutputs         = 3;
sizes.NumInputs           =5;
sizes.DirFeedthrough = 1;
sizes.NumSampleTimes = 1;
sys=simsizes(sizes);

x0=0*(1:10);
```

```
str=[];

ts=[-1 0];

hidden3=9;b3=10;c3=1.01;

function sys=mdlDerivatives(t,x,u)

global hidden3 c3 b3;

x1d=u(1);

x3d=u(2);

x3_bar=u(3);

x2=u(4);

x3=u(5);


rou3=0.05;epc=0.01;


tol3=0.01;

S3=x3-x3d;

dx3d=(x3_bar-x3d)/tol3;


xx=[x2;x3];


d3=[-9 -7 -5 -3 0 3 5 7 9;

    -9 -7 -5 -3 0 3 5 7 9];


for i=1:hidden3

    kesi3(i)=exp(-((norm(xx-d3(:,i)))^2)/(2*b3^2));

end

fai3=[kesi3,rou3^2*S3/(2*epc)-dx3d+c3*S3]';

eta3=0.001;
```

```
gama3=20*eye(10);

dth3=gama3*S3*fai3-gama3*eta3*x;
for i=1:hidden3+1
    sys(i)=dth3(i);
end

function sys=mdlOutputs(t,x,u)
global hidden3 c3 b3;
x1d=u(1);
x3d=u(2);
x3_bar=u(3);
x2=u(4);
x3=u(5);

rou3=0.05;epc=0.01;

tol3=0.0198;
S3=x3-x3d;
dx3d=(x3_bar-x3d)/tol3;

xx=[x2;x3];
d3=[-9 -7 -5 -3 0 3 5 7 9;
    -9 -7 -5 -3 0 3 5 7 9];

for i=1:hidden3
```

```
    kesi3(i)=exp(-((norm(xx-d3(:,i)))^2)/(2*b3^2));
end
fai3=[kesi3,rou3^2*S3/(2*epc)-dx3d+c3*S3]';

th3=x;
th3_w=x(1:9);

sys(1)=x(10);          %1/a3
sys(2)=th3_w'*kesi3'; %to approximate 1/a3*f3
sys(3)=-th3'*fai3;     %u


%%%%%%%%%%%%%%%%%%%%%%%%%ADSC x3 Bar
function [sys,x0,str,ts]=s_function(t,x,u,flag)
switch flag,
case 0,
    [sys,x0,str,ts]=mdlInitializeSizes;
case 1,
    sys=mdlDerivatives(t,x,u);
case 3,
    sys=mdlOutputs(t,x,u);
case {2, 4, 9 }
    sys = [];
otherwise
    error(['Unhandled flag = ',num2str(flag)]);
end
function [sys,x0,str,ts]=mdlInitializeSizes
```

```
global hidden2 c2 b2;
sizes = simsizes;
sizes.NumContStates    = 9;
sizes.NumDiscStates    = 0;
sizes.NumOutputs          = 2;
sizes.NumInputs            =4;
sizes.DirFeedthrough = 1;
sizes.NumSampleTimes = 1;
sys=simsizes(sizes);

x0=0*(1:9);
str=[];
ts=[-1 0];
hidden2=9;b2=10;c2=2.01;
function sys=mdlDerivatives(t,x,u)
global hidden2 c2 b2;
x1=u(1);
x2=u(2);
x2d=u(3);
x2_bar=u(4);

S2=x2-x2d;
tol2=0.01;
dx2d=(x2_bar-x2d)/tol2;

xx=[x1;x2];
```

```
d2=[-9 -7 -5 -3 0 3 5 7 9;
    -9 -7 -5 -3 0 3 5 7 9];

for i=1:hidden2
    kexi2(i)=exp(-((norm(xx-d2(:,i)))^2)/(2*b2^2));
end
fai2=kexi2';

eta2=0.002;
gama2=30*eye(9);

dth2=gama2*fai2*S2-gama2*eta2*x;
for i=1:hidden2
    sys(i)=dth2(i);
end

function sys=mdlOutputs(t,x,u)
global hidden2 c2 b2;
x1=u(1);
x2=u(2);
x2d=u(3);
x2_bar=u(4);

rou2=0.1;epc=0.01;

S2=x2-x2d;
tol2=0.0198;
```

```
dx2d=(x2_bar-x2d)/tol2;

xx=[x1;x2];
d2=[-9 -7 -5 -3 0 3 5 7 9;
    -9 -7 -5 -3 0 3 5 7 9];

for i=1:hidden2
    kesi2(i)=exp(-((norm(xx-d2(:,i)))^2)/(2*b2^2));
end
fai2=kesi2';

th2=x;

sys(1)=-th2'*fai2-rou2^2*S2/(2*epc)+dx2d-c2*S2; %x3_bar
sys(2)=th2'*fai2;    %f2

%%%%%%%%%%%%%%%%%% Low_filter1
function [sys,x0,str,ts]=s_function(t,x,u,flag)
switch flag,
case 0,
    [sys,x0,str,ts]=mdlInitializeSizes;
case 1,
    sys=mdlDerivatives(t,x,u);
case 3,
    sys=mdlOutputs(t,x,u);
case {2, 4, 9 }
    sys = [];
```

```
otherwise
    error(['Unhandled flag = ',num2str(flag)]);
end
function [sys,x0,str,ts]=mdlInitializeSizes
sizes = simsizes;
sizes.NumContStates   = 1;
sizes.NumDiscStates   = 0;
sizes.NumOutputs       = 1;
sizes.NumInputs        =1;
sizes.DirFeedthrough = 1;
sizes.NumSampleTimes = 1;
sys=simsizes(sizes);
x2b0=0;
x0=[x2b0];
str=[];
ts=[-1 0];
function sys=mdlDerivatives(t,x,u)
tol2=0.0198;
x2_bar=u(1);

sys(1)=1/tol2*(x2_bar-x(1));
function sys=mdlOutputs(t,x,u)
sys(1)=x(1);   %x2d


%%%%%%%%%%%%%%% Low_filter2
function [sys,x0,str,ts]=s_function(t,x,u,flag)
switch flag,
```

```
case 0,
    [sys,x0,str,ts]=mdlInitializeSizes;
case 1,
    sys=mdlDerivatives(t,x,u);
case 3,
    sys=mdlOutputs(t,x,u);
case {2, 4, 9 }
    sys = [];
otherwise
    error(['Unhandled flag = ',num2str(flag)]);
end
function [sys,x0,str,ts]=mdlInitializeSizes
sizes = simsizes;
sizes.NumContStates    = 1;
sizes.NumDiscStates    = 0;
sizes.NumOutputs          = 1;
sizes.NumInputs           =1;
sizes.DirFeedthrough = 1;
sizes.NumSampleTimes = 1;
sys=simsizes(sizes);
x3b0=0;
x0=[x3b0];
str=[];
ts=[-1 0];
function sys=mdlDerivatives(t,x,u)
tol3=0.0198;
x3_bar=u(1);
```

```
sys(1)=1/tol3*(x3_bar-x(1));

function sys=mdlOutputs(t,x,u)

sys(1)=x(1);    %x3d


%%%%%%%%%%%%%%%%%%%%%%LSTM

clear,clc

data = importdata('S1.txt');%%%%%%%%%

data = data';

figure()

plot(data,LineWidth=5)



%%%%%%%%%%%%%%%%%%%%%%%%

% Define the test and training length

numTimeStepsTrain = floor(0.9*numel(data));

dataTrain = data(1:numTimeStepsTrain+1);

dataTest = data(numTimeStepsTrain+1:end);


%%%%%%%%%%%%%%%%%%%%%%%% Data normalization In order to better match and prevent training divergence, the training data is normalized to zero mean and unit variance.

% When forecasting, you must use the same parameters as the training data to standardize the test data.

% defines the test and training length.

% training data normalization

mu = mean(dataTrain);

sig = std(dataTrain);
```

```
dataTrainStandardized = (dataTrain - mu) / sig;


XTrain = dataTrainStandardized(1:end-1);

YTrain = dataTrainStandardized(2:end);


%%%%%%%% Define the network structure.

%% Create LSTM regression network. The LSTM layer is designated as having 128 hidden cells.

% define the network structure

layers = [

    sequenceInputLayer(1,"Name","input")

    lstmLayer(128,"Name","lstm")

    dropoutLayer(0.2,"Name","drop")

    fullyConnectedLayer(1,"Name","fc")

    regressionLayer];

options = trainingOptions('adam', ...%Adam

    'MaxEpochs',250, ...

    'GradientThreshold',1, ...

    'InitialLearnRate',0.001, ...

    'LearnRateSchedule','piecewise', ...

    'LearnRateDropPeriod',125, ...,

    'LearnRateDropFactor',0.2, ...

    'Verbose',0, ...

    'Plots','training-progress');


%%%%%%%%%%%%%%
```

%% use TrainNetwork to train LSTM network.

% training network

net = trainNetwork(XTrain,YTrain,layers,options);

%%%%%%%% Forecast

%% To predict the values at multiple future time points, use the forectAndUpdateState function to predict one time point at a time, and update the network status each time. For each prediction, the previous prediction is used as the input of the function.

% use the same parameters as the training data to standardize the test data.

dataTestStandardized = (dataTest - mu) / sig;

XTest = dataTestStandardized(1:end-1);

net = predictAndUpdateState(net,XTrain);

[net,YPred] = predictAndUpdateState(net,YTrain(end));

numTimeStepsTest = numel(XTest);

for i = 2:numTimeStepsTest

    [net,YPred(:,i)] = predictAndUpdateState(net,YPred(:,i-1),'ExecutionEnvironment','cpu');

end

% In order to initialize the network state, the training data XTrain is predicted first. Next, the last time step of the training response YTrain is used for the first prediction (end). Loop through the remaining predictions and input the previous predictions into the forectAndUpdateState.

% For large data sets, long sequences or large networks, it is usually faster to calculate predictions on GPU than on CPU. Otherwise, the calculation speed of prediction on CPU is usually faster. For single time step prediction, please use CPU. To use CPU for prediction, please set the %ExecutionEnvironment' option of recrectAndUpdateState to' CPU'.

% Use the previously calculated parameters to denormalize the forecast.

```
% drawing
figure
plot(dataTrain(1:end-1))
hold on
idx = numTimeStepsTrain:(numTimeStepsTrain+numTimeStepsTest);
plot(idx,[data(numTimeStepsTrain) YPred],'k.-'),hold on
plot(idx,data(numTimeStepsTrain:end-1),'r'),hold on
hold off
xlabel("Time",'linewidth',3)
ylabel("Data",'linewidth',3)
% title("Forecast")
h1=legend(["Observed" "Forecast"])
set(h1,'Interpreter','latex','fontsize',16);


net = resetState(net);
net = predictAndUpdateState(net,XTrain);


% Reset network status
net = resetState(net);
net = predictAndUpdateState(net,XTrain);
%

YPred = [];
numTimeStepsTest = numel(XTest);
```

```
for i = 1:numTimeStepsTest
    [net,YPred(:,i)] = predictAndUpdateState(net,XTest(:,i),'ExecutionEnvironment','cpu');
end

YPred = sig*YPred + mu;

%
figure
plot(dataTrain(1:end-1),'linewidth',3)
hold on
idx = numTimeStepsTrain:(numTimeStepsTrain+numTimeStepsTest);
plot(idx,[data(numTimeStepsTrain) YPred],'k.-','linewidth',3),hold on
plot(idx,data(numTimeStepsTrain:end-1),'r','linewidth',3),hold on
hold off
xlabel("Time",'linewidth',3)
ylabel("Data",'linewidth',3)
% title("Forecast")
h1=legend(["Observed" "Forecast"])
set(h1,'Interpreter','latex','fontsize',16);
```
